# Hospital and Institutional News

**Published:** 1916-12-23

**Authors:** 


### HOSPITAL AND INSTITUTIONAL NEWS.

### DEATH OF SIR FREDERIC EVE.

The surgical world and the London Hospital have lost, by the death of Lieut.-Colonel Sir Frederic Eve, one who worthily maintained the best traditions of both. Since the war began Sir Frederic has been consulting surgeon to the Eastern Command, with the rank of Lieutenant-Colonel in the Royal Army Medical Corps. His life work, however, by which he will be best remembered, lay in his surgical work at the London, where he was senior surgeon for some time, until made consulting surgeon in 1914, and in his activities as a teacher there. It would be idle to class Eve amongst the pioneer research workers in surgery. He made no revolutionary discovery or invention, and established no new principle. But, for all that, he was always eager and able to see the merits of new ideas when they were sound, as well as the flaws in those that were unsound; and he was never one who allowed himself to get fossilised in the practice of his art. Educated at St. Bartholomew's, whence he qualified in 1876, and took the F.R.C.S. in 1878, Sir Frederic Eve first turned his attention to pathology. He was curator of the museum of pathology at St. Bartholomew's, and then of that of the Royal College of Surgeons, in the early 'eighties; and many of his later contributions to surgical literature displayed his intimate knowledge of pathology. Besides his appointment at the London Hospital, Sir Frederic was also for many years on the staff of the Evelina Hospital for Children, and was consulting surgeon to that institution. He was a valued member of the Council of the Royal College of Surgeons, and at the time of his death was a vice-president of that body.

### NEW SECRETARY TO THE MIDDLESEX HOSPITAL.

The vacancy in the office of Secretary-Superintendent of the Middlesex Hospital, created by the sudden death of Mr. F. Clare Melhado, who served the hospital so devotedly for thirty-seven years, has been filled by the appointment of Mr. Walter Kewley. Mr. Kewley entered the service of the Middlesex Hospital in January 1906, and as Resident Assistant Secretary was intimately associated with the late Mr. Melhado in the general management of the hospital, and in the conduct of the many schemes for its extension and improvement which have been brought to successful fulfilment during the past ten years. In earlier life Mr. Kewley had experience in civil, electrical, and mechanical engineering, and also spent some years as a tutor. The knowledge he thus acquired has proved of considerable value in connection with his work at the hospital. Eleven years' service in a position of high responsibility in a great hospital like the Middlesex is a genuine and stern test of the aptitudes and qualifications of any man for hospital work of the best. In these circumstances, Mr. Walter Kewley's appointment by the Governors of the Middlesex Hospital to succeed Mr. Melhado is the most valuable testimonial possible to his qualifications and claims for the post. We could wish that hospital committees throughout the country would spend a little more money on salaries, so as to enable them to select, appoint, and train men capable to succeed, when found competent, to the highest post in their gift. The call for men of education, high character, and marked ability for hospital work was never more urgent than it is to-day, though the search for them, when an important post becomes vacant under existing conditions, seldom yields an adequate choice of candidates of the highest capacity and fitness. We therefore congratulate the authorities of the Middlesex Hospital upon the election of Mr. Walter Kewley, and upon the wisdom of the policy which has placed him at their disposal in the grave crisis caused by the sudden death of Mr. Clare Melhado. We wish many years of increased usefulness and success to Mr. Kewley in his new office, and hope he may have the best of health to pursue and enjoy the greater responsibilities which will now devolve upon him.

### HANDSOME NEW ANNUAL SUBSCRIPTIONS.

A number of institutions have, during the last few days, received substantial support, which it is hoped will be renewed annually, under the will of the late Sir Sigmund Neumann, Bart. At the request of Sir Sigmund the family have arranged to continue to support the charities he was accustomed to subscribe to, and for the purpose the income of £100,000 will be set aside. Distribution of this income has commenced, and we believe that amongst the allocations made, of which a list has not yet been published, is one of £100 to Queen Charlotte's Hospital, and others of £50 each to the Hospital for Epilepsy, Maida Vale, and the Hospital for Diseases of the Chest, Victoria Park. The executors are the testator's brother, Mr. Ludwig Neumann, Mr. J. L. Bergson, and the Public Trustee. The name of the latter has in recent years appeared frequently in a similar capacity; if many charitable bequests, as in this case, are to be distributed at his discretion he will be a much-sought-after man. New annual subscriptions of £50 or £100 are not picked up easily in these days when heavy taxation and expenses do not permit many people to promise any part of their future income.

### A NEW APPOINTMENT.

At a special meeting of the board of the Leicester Royal Infirmary, Dr. W. W. Mackarell, from the University of Liverpool, was elected to the post of pathologist for an initial period of one year at a salary of £500 per annum. The appointment is an important one in view of the increasing recognition of the importance of pathology in medical and surgical treatment, and of the large share that this branch of medical knowledge will have in the national treatment of venereal disease under the Local Government Board scheme. Dr. Mackarell is a Doctor of Medicine and a D.P.H. of the Liverpool School of Medicine, and holds the Diploma of Tropical Medicine of the same University. He has held many appointments in his special branch of medicine.

### PRESENTATION TO A HOSPITAL CHAIRMAN.

Mr. T. B. Jones, the chairman of the finance committee of the Loughborough Hospital and Dispensary, was the recipient of an interesting presentation on his departure to Carlisle to take up an important banking preferment. Alderman Mayo, under whose auspices the presentation was made, presided; and Mr. W. B. Paget, the president of the hospital, in asking Mr. Jones' acceptance of a solid silver salver of Georgian design and a silver tea-kettle and stand, referred to the important services which Mr. Jones had rendered to the hospital. He had found him a most valuable helper, and of the greatest possible assistance on matters of hospital finance, his exposition of which had been particularly lucid, and showed the keen grasp he had of the position of affairs. The presentation committee desired to associate Mrs. Jones with the gift by asking her acceptance of a sum remaining in hand, with which they hoped she would purchase a personal gift. The Mayor of Loughborough also paid tribute to Mr. Jones' services to the town as borough treasurer and in numerous other capacities. Mr. Jones, in response, alluded to the growth of the borough in recent years and to the joy it had been to him to be of service to the hospital.

### CHARGES AGAINST A WALSALL PRACTITIONER UPHELD.

Some time ago a series of charges was brought against a doctor at Walsall of improper conduct as chairman of the Walsall Insurance Committee and in other respects. The behaviour attributed to this doctor was so extraordinary that at the time it seemed incredible that anyone in his position should so misuse his authority, for which reason no mention was then made of the case in The Hospital. However, the National Health Insurance Commissioners have held an inquiry into the allegations, and their report severely condemns Dr. J. J. Lynch, and removes him from the panel list. This practitioner is a borough magistrate, a member of the Town Council and of the Education Committee, and was chairman of the Walsall Insurance Committee. The Commissioners find that he utilised the latter position and that of member of other committees to limit the number of patients accepted by other panel practitioners—his rivals in practice; that he improperly took into his own hands the allocation of persons who had not chosen a medical attendant, and conducted the allocation with a view to his own private advantage; and that he endeavoured by improper means to stifle two complaints made against him by panel patients. The attention of the General Medical Council may well be directed to these findings and to the evidence by which they are supported. The case, we would fain believe, is unique; but it shows that there are opportunities of crooked dealing under the panel system, and possibly explains some of the smouldering discontent among doctors who work for Insurance Committees.

### WOMEN RESIDENTS AT THE LONDON HOSPITAL.

According to a statement in a provincial paper, which we have not noticed in the London press, the authorities of the London Hospital have decided to accept qualified medical women for some of the resident appointments there. If this statement is true, and there seems no reason why it should not be, the time is not likely to be distant when many other leading hospitals which have not yet admitted women to the resident medical staff will be obliged to do so. For if the London Hospital, with its great school and its established *prestige*, cannot secure enough men to fill these posts, the same difficulty (in a more acute form possibly) is certain to exist elsewhere. The reason for this dearth of male residents is, it need scarcely be said, the demand made upon all young qualified doctors to serve abroad in the Royal Army Medical Corps. The logical extension of the principle at stake is the admission of women to compete on equal terms with men for the visiting staff appointments at this—or any other—hospital. Such a development is conceivably in store some day; but can hardly be regarded as likely at present, even in war-time, at any hospital where the medical school is confined to male students.

### DOCTORS' SALARIES IN IRELAND.

There appears to be a tendency in some parts of Ireland to increase the salaries of district medical officers. Those in the service of the Carlow Union have been raised, and the increase, in spite of some criticism, has been made retrospective. The maximum salary for the medical officer of Carlow Dispensary is now £212. It is noteworthy that the critics include the representatives of the farmers, whose present "good time" is being explained away on the ground that they are expected to disburse their recent profits as soon as they have pocketed them. Probably the broad truth is that district medical officers, in Ireland especially, have long been underpaid, and that the reform of this abuse now forced on the local authorities is not being carried out cheerfully or without demur.

### OFFICERS AS OUT-PATIENTS.

The British Red Cross Society and the Order of St. John have now enlarged the out-patient clinic for officers which was lately established at 126 Great Portland Street. Elaborate bath-treatments are provided, including the now fashionable whirlpool bath, which precedes manipulation of joints and muscles. Electric treatment is also given. The attendances are understood to be regular, and the results satisfactory. There is sufficient accommodation for about 100 attendances a day. The officer out-patient appears to appreciate the clinic, and, we hope, does not experience those waiting hours which have seemed in the past unavoidable at the big hospitals.

### SECRET CRITICS OF THE WELSH MEMORIAL ASSOCIATION.

The Swansea Corporation Health Committee lately criticised the accounts of the King Edward Memorial Association on the ground that the balance-sheet had never been signed. Indeed, one speaker, the chairman of the health committee, Mr. W. Owen, declared that the balance-sheet had had to be recalled, and a Treasury audit made. He went on to say that while some local authorities made contributions out of the rates to the Association, others were paying according to the number of "users." He added that the Treasury audit showed the basis of the contributions received, while the Association's statement did not. It was decided, on the motion of the chairman, to pay no further calls until the committee received a copy of the Treasury audit. The suggestion underlying the chairman's criticism is that the Association has not been very scrupulous in its methods of securing those contributions from the rates which, according to him, it desires. The matter should be easily settled, and the Association is entitled also to a hearing. But we mention the matter not so much for its own sake as because, when criticisms of the Association are made, which has not been infrequent, one medical man or another writes from Wales and either makes sarcastic comments on the proceedings or asks us to investigate the matter. These correspondents, though they sign their names, are always careful to mark their letters "Not for publication," and when pressed, as we have sometimes pressed them, to state their ground of complaint, if they have any, they have invariably proved unwilling to pursue the matter publicly. Now this, in our view, is as much open to criticism as the proceedings of the Association may have been. We therefore ask, with some impatience, that if the medical men in Wales wish to criticise the Association they should state their grievance openly, and at least be men enough to mention what it is that they wish to be "investigated."

### MR. W. CROOKS AS AN IN-PATIENT.

The Poplar Hospital for Accidents has had the satisfaction of attending to Mr. Will Crooks, whose gratitude to the whole staff has been shared and expressed by Mrs. Crooks in a letter addressed to the institution. The patient has also contributed £10 to the appeal now necessitated by the financial anxieties of the hospital. This testimonial from one who knows the East End and the labour world as well as Mr. Crooks should do much to make the classes from which the patients of the Poplar Hospital are drawn make a special effort to raise the money wanted. Since these classes include the employers also there can be no reason why the money should not be promptly raised.

### A BIG HOSPITAL FOR GERMAN PRISONERS.

A large hospital for German wounded is being opened near London at an early date. It will accommodate 1,200 German prisoners, and, incidentally, relieve certain local authorities from what they find an ungrateful task—namely, accepting or refusing to accommodate such patients along with British wounded in the same institution. The Mile End Board of Guardians has indeed refused to do so, and, since it is better that no controversy should arise over the matter, we hope that the new institution will be ready at an early date. In view of the Allied successes on the Western front there can be no guarantee that still another big hospital may not soon be wanted.

### THE BAKER STREET BAZAAR.

The Portman Rooms, described above under their old and indeed historic title, have now been appropriated by the Government as a military hospital in connection with the hospital for officers which has been established at the Great Central Hotel. The Press has reminded the world that the history of these rooms is curious. Used as a military barracks one hundred years ago, later the home of Madame Tussaud, later still the place in which was held the annual Cattle Show, since housed in the Agricultural Hall, they became not only the Baker Street Bazaar, but eventually the Portman Rooms for fashionable gatherings and dances. Coming thus under the eye of the Office of Works an additional exit was constructed, and this, according to the *Times*, was the tunnel alluded to in the Druce case as that through which, on Mrs. Druce's showing, the fifth Duke of Portland used to pass when, in the disguise and habit of Mr. T. C. Druce, he represented himself as the proprietor of the bazaar in Baker Street. The cycle of change is complete with their century of existence, and so we find a reversion at last, not indeed to a military barracks, but aptly enough to a military hospital.

### TRINITROTOLUENE POISONING.

The Ministry of Munitions has taken a sensible and a courageous step in compiling and issuing to such as it may concern a memorandum dealing with what is colloquially known as T.N.T. poisoning. Partly through injudicious publicity in the press, partly by the activity of the pessimist gossipmonger type of individual, there has been a distinct scare amongst the public as to the danger of working with and handling this explosive. The memorandum sets matters in their true light: neither minimising the risks of T.N.T. poisoning, nor exaggerating them. The result shows how minute the danger really is, and how by due precautions that minuteness may be reduced practically to vanishing point. At the same time much valuable information is given about the symptoms, diagnosis, and treatment of T.N.T. and D.N.B. (di-nitro-benzene) poisoning, as well as on the more important matter of prophylaxis. The precautions adopted at the Royal Arsenal may serve as a model. They include: the employment of those alone who are in good general health, and as far as practicable between twenty and fifty years of age; the wearing of special clothing, and of veils, respirators, and gauntletted gloves; inspection by a medical officer once a week; fortnightly alterations of employment; mechanical devices for preventing dust and getting rid of fumes; adequate ventilation of workshops; facilities for getting suitable and sufficient food, including the provision of milk free to the workers on their arrival; and compulsory washing before meals and before leaving the factory, with neutral soap and individual towels.

### TREATMENT.

When the disease has developed, or is even suspected, the treatment recommended depends on whether or not jaundice is present. If this symptom has not appeared, removal from all possibility of contact with the source of the trouble and one or two days' rest in bed is usually enough; the diet is restricted to milk, milk puddings, fruit, green vegetables, barley water, lemonade, tea and coffee. Vegetable laxatives are given, and also a mixture containing sodium sulphate, potassium citrate, and sodium bicarbonate. When jaundice is present the outlook is much more serious, and absolute rest in bed is essential. Milk diet is ordered, increasing up to four pints a day. White mixture is given regularly; and alkali-producing drugs, such as citrates and bicarbonates, are given to counteract the tendency to acid intoxication. Rectal and intravenous salines are also useful in severe cases. In an addendum to this official memorandum, details are given of a new test for the early diagnosis of those who are suffering from the first symptoms of T.N.T. absorption. This test, if it bears out the expectations expressed in the memorandum, should prove of great assistance in detecting the early, that is curable, cases, and in preventing thereby the development of serious cases. The whole pamphlet is excellent in manner and matter, and should go far to allay public anxiety about this disease.

### THE COCAINE AND OPIUM REGULATIONS AND THEIR EFFECT ON INSTITUTIONS.

The amended regulations issued on December 5 regarding the use and supply of "cocaine" (which includes all preparations, salts, derivatives, or admixtures prepared therefrom or therewith and containing one part in one thousand or more of the drug) would appear to affect very closely the working of our hospitals and similar institutions. The original Order of July 28 stated that "persons entitled" to carry on the business of a chemist and druggist were, as such, "authorised persons" under the Order to dispense and supply cocaine and its preparations to the prescription of a medical practitioner. The amended Order of December 5, however, reads, "authorised person—a person carrying on the retail business of a chemist and druggist." The remarkable position now seems to obtain that at all our hospitals, infirmaries, etc., when the pharmacist dispenses cocaine solution and solid or semi-solid preparations of opium, the law is contravened unless a special permit is obtained from the Home Office making the responsible official (the pharmacist) a duly "authorised" person. It is certainly a curious anomaly that the hospital pharmacist is not, by virtue of his qualification, placed in an equal position to that of his *confrère* who keeps a chemist's shop. An effect of the above Order will be that, unless the conditions of the Amended Order are revised, the Home Office will have to deal with an application from every hospital in the Kingdom for a permit. Medical practitioners in busy hospital practice will also find the new Order gives them added work; thus, every medical practitioner is required to enter on any prescription for cocaine his full name, address, and qualifications, also marking the prescription with the words "Not to be repeated" and the date. A noteworthy feature of the new Order as to labelling cocaine preparations is that now hospital out-patients when given, say, "cocaine eye-drops," are aware of the constituents, the quantity having to be stated on the label; whether this is desirable is open to question. It would have been much better to have excluded hospitals and bona-fide institutions for the treatment of the sick and infirm from the provisions of the Order.

### A UNIQUE CHAPEL.

It will be remembered that some time ago we published in these columns a short article dealing with the various phases of the work of the St. Dunstan's Home for Blinded Soldiers. We now learn that a welcome addition has been made to the institution by way of a chapel for the patients and staff. The building has been erected in the grounds of the home, and an assistant chaplain has been appointed. The Hon. Chaplain is the Vicar of Holy Trinity, Marylebone.

### THIS WEEK'S DRUG MARKET.

As is to be expected, business in the drug market is quiet and will probably continue so until early in the New Year, when the process of stock-taking will be completed. In the meantime, buyers are restricting their purchases to their immediate requirements. Of noteworthy price changes there are few, but the general tendency of values is still in an upward direction, although in one or two instances there have been recessions. Acetylsalicylic acid is again slightly cheaper, and it is not improbable that the gradual downward movement will continue, although no substantial drop is expected. Salicylic acid and sodium salicylate are unchanged, but here again it is probable that the lowest figures have not yet been reached. Amidopyrin is slightly lower in price and the same may be said of phenazone. Barbitone and phenacetin continue scarce, but supplies are expected to arrive from the United States of America shortly, when we may see somewhat lower values; for the present, however, purchasers will have to continue to pay extreme prices. A small business has been done in quinine and prices are fairly well maintained. Potassium permanganate is scarcer than ever, and extraordinary prices are quoted. Bromides are unchanged. The high value of opium is well maintained. Resorcin is difficult to obtain.

